# Food Insecurity, Financial Hardship, and Mental Health Among Multiple Asian American Ethnic Groups: Findings from the 2020 COVID-19 Household Impact Survey

**DOI:** 10.1089/heq.2021.0179

**Published:** 2022-06-24

**Authors:** Jessica Y. Islam, Iman Awan, Farzana Kapadia

**Affiliations:** ^1^ Cancer Epidemiology Program, H. Lee Moffitt Cancer Center and Research Institute, Tampa, Florida, USA.; ^2^ Morsani College of Medicine, University of South Florida, Tampa, Florida, USA.; ^3^ Department of Epidemiology, School of Global Public Health, New York University, New York, New York, USA.

**Keywords:** food insecurity, financial hardship, COVID-19 pandemic, Asian Americans, anxiety, hopelessness, disaggregated data, disparities

## Abstract

**Background:**

The COVID-19 pandemic has adversely impacted the financial and mental well-being of U.S. adults, however, Asian American (AA)-specific data are lacking, particularly disaggregated by AA ethnicity. Our objective was to evaluate food insecurity (FI), financial hardship, and mental health among disaggregated AA ethnic groups during the COVID-19 pandemic.

**Methods:**

We used data from the COVID-19 Household Impact Survey, a sample of 10,760 U.S. adults weighted to reflect the U.S. population (*weighted n: 418,209,893)*. AA ethnic categories were based on self-report (*n*=312, 5.1%; *weighted n: 21,143,079*) and provided as follows: Chinese American, South Asian, Filipino+Vietnamese, and Japanese+Korean. We estimated the prevalence of FI and financial hardship across AA ethnic categories. We estimated the demographic determinants of FI, including financial hardship, among AA adults using multivariable Poisson regression. We calculated the prevalence of mental health symptoms among food-insecure AA adults, as well as among AA adults experiencing both FI and financial hardship.

**Results:**

Overall, the prevalence of FI and financial hardship among AA adults was highest among Filipino+Vietnamese adults (52.9–24.5%) and lowest among Japanese+Korean adults (13.9–8.6%). Determinants of FI among AA adults included Filipino+Vietnamese ethnicity (adjusted prevalence ratios [aPR]: 2.81, 95% confidence interval [CI]: 1.49–5.29), being widowed/divorced/separated (aPR: 3.14, 95% CI: 1.37–7.23), high school graduate only (aPR: 3.46, 95% CI: 1.96–6.11), having low income <$30,000 (aPR: 2.54, 95% CI: 1.27–5.06), and living in rural areas (aPR: 7.65, 95% CI: 1.17–50.14). Eighty-one percent and 63% of AA adults with anxiety and hopelessness at least 3–7 days/week, respectively, were food insecure and experiencing financial hardship.

**Conclusion:**

Disparities exist in FI and financial hardship among AA adults, particularly Filipino+Vietnamese adults, and are associated with increased self-reporting of feelings of anxiety and hopelessness.

## Background

In the United States, the COVID-19 pandemic has led to not only a public health crisis but also an economic crisis due to several complicated and overlapping factors, including mandatory business closures, record high unemployment rates, recommended COVID-19 preventive behaviors such as social distancing or quarantine, increase cost of goods due to supply-chain disruptions, and increased demand for living space as more adults worked from home.^1^ During the second quarter of 2020, the economy shrank a record of 31%,^2^ which may lead to an estimated net U.S. GDP loss ranging from $3.2 trillion (14.8%) to $4.8 trillion (23.0%) in a 2-year period.^1^ The economic impact of the pandemic has disproportionately affected the racial/ethnic minority groups. Existing inequities in social determinants of health, including income or wealth, educational attainment, employment opportunities, race-based discrimination, and housing, were exacerbated during the pandemic.

For example, in 2020, an estimated 72% of Hispanic or Latinx, 61% of Black, and 55% of Native American adults reported experiencing serious financial problems during the coronavirus outbreak.^3^ Rates of food insecurity (FI), a condition akin to hunger when a household does not have sufficient food to feed its members and lacks the resources to obtain more food, grew to 32% of households in July 2020, with the highest burdens among the Black and Hispanic households.^4^ Other state-specific surveys found that Black, Asian, and Hispanic/Latinx adult respondents were similarly likely to report that a member of their household lost a job, was placed on furlough, or had work pay or hours reduced due to the pandemic,^5^ suggesting that Asian Americans (AAs) have also been adversely financially impacted by the pandemic.

The AA racial group has the highest income inequality compared with the other racial/ethnic groups in the United States.^6^ Income inequality, a measure of the economic gap between the highest and lowest income groups, has increased over time at the highest rate for the AA population over the past 50 years.^6^ However, the experiences of low-income AAs are frequently overlooked due to stereotypes such as the “model minority” and the implicit bias related to the overall higher educational attainment and higher income levels among AAs.^7^ As highlighted by the implementation of the White House Initiative for AAs and Pacific Islanders,^8^ an understanding of the disparate outcomes across AA ethnicity requires acquiring insights into different trends in socioeconomic status across disaggregated Asian ethnicities.

For example, data from the American Community Survey demonstrate that when compared with white adults, South Asian, Japanese, and Filipino adults are less likely to experience poverty, and Chinese, Korean, and Vietnamese adults are more likely to live in poverty.^9^ In the context of health, a more recent national study demonstrated that socioeconomic status, including household income, played a mediating role in reduced self-rated health among Cambodian, Chinese, Hmong, and Vietnamese Americans, but not for Korean and Filipino Americans.^10^ Given that the low-income families of color with children were most likely to experience an income shock and FI during the pandemic,^4^ it is particularly important to investigate the differential impacts among AA ethnic groups.

Experiences such as financial hardship and FI have been associated with poor mental health outcomes during the COVID-19 pandemic in the general U.S. population. For example, based on data from the United States Department of Agriculture (USDA) Household Food Security Module conducted in March 2020, FI was associated with screening positive for depression and high perceived stress.^11^ However, research evaluating the potential mental health effects of socioeconomic stressors in the context of the pandemic among AAs has been limited and existing research has focused on the prevalence of FI among AAs as a whole. For example, recent data suggest that although Asian households are less likely to experience FI compared with other racial/ethnic minority groups, these households have grown significantly less confident in their anticipated household food security during the COVID-19 pandemic due to limited transportation, mobility, and health issues.^12^

Furthermore, during the COVID-19 pandemic, Asian food insecure adults reported feeling afraid to go out to buy food,^2^ which may be attributed to the rise of xenophobia toward AAs during the COVID-19 pandemic.^13^ Investigating the heterogeneity in mental health outcomes in the context of FI and financial hardship within and across AA ethnic groups is imperative due to the differential experiences across AA ethnicities based on income and wealth, essential worker status, and discrimination.

Thus, our goal was to examine the experiences of FI with financial hardship and mental health outcomes across distinct Asian ethnic groups using data from the COVID-19 Household Impact Survey. We hypothesized there will be significant disparities in the prevalence of FI and financial hardship across disaggregated AA ethnic groups. Furthermore, we hypothesized that FI and financial hardship will be associated with mental health symptoms. As we were unable to disentangle the temporal relationship between FI and financial hardship due to the cross-sectional nature of the study, we explored these two social phenomena independently and as concomitant conditions.

## Methods

### COVID-19 impact survey

Data for these analyses were obtained from the publicly available COVID-19 Household Impact Survey, conducted by National Opinion Research Center (NORC) at the University of Chicago. Ethical approval was waived given the data collected through the COVID-19 Household Impact Survey are publicly available and are not considered human subjects research. The COVID-19 Household Impact Survey provides national and regional statistics on physical health, mental health, economic security, and social dynamics of the U.S. population,^14^ identified through the AmeriSpeak^®^ sample. The survey is designed to provide weekly estimates of the U.S. adult household population nationwide. Cross-sectional data from week-1 (April 20–26, 2020), week-2 (May 4–10, 2020), and week-3 (May 30–June 8, 2020) were merged for the present analysis.

### AmeriSpeak sample

Funded and operated by NORC at the University of Chicago, AmeriSpeak is a probability-based panel designed to be representative of the U.S. household population. During the initial recruitment phase of the AmeriSpeak panel, randomly selected U.S. households were sampled using area probability and address-based sampling, with a known, nonzero probability of selection from the NORC National Sample Frame. The panel provides sample coverage of ∼97% of the U.S. household population. Those excluded from the sample include people with P.O. Box-only addresses, some addresses not listed in the U.S. Postal Service Delivery Sequence File, and some newly constructed dwellings. AmeriSpeak panelists participate in NORC studies or studies conducted by NORC on behalf of governmental agencies, academic researchers, and media and commercial organizations.

Interviews were conducted in English and Spanish, representing the 50 U.S. states. Panel members were randomly drawn from AmeriSpeak. In households with more than one adult panel member, only one was selected at random for the sample. Invited panel members were given the option to complete the survey online or by telephone with an NORC telephone interviewer. The number of participants invited and the percentage of interviews completed by week are as follows: 11,133 invited with 19.7% interviews completed during week 1; 8570 invited with 26.1% interviews completed during week 2; and 10,373 invited with 19.7% interviews completed during week 3. The survey sample includes 10,760 adults nationwide. The final analytic sample was weighted to reflect the U.S. population of adults aged 18 years and older. Demographic weighting variables were obtained from the 2018 American Community Survey. Further details regarding NORC's weighting methodology can be found here: www.covid-impact.org/results.

### AA study sample

The COVID-19 Household Impact Survey provides ethnicity-based categories for participants who self-identify as non-Hispanic Asian disaggregated into the following Asian ethnic groups: Chinese, South Asian, Filipino or Vietnamese, and Japanese or Korean. Results are presented for the total study sample (all race/ethnicities), the aggregated non-Hispanic AA sample, and the disaggregated AA ethnic groups identified above. Race/ethnicity was suppressed for 1.5% of the study sample due to the potential for disclosure risk; these observations were dropped from the present analysis.

### Primary measures

Our primary measures for this analysis were FI, financial hardship, and mental health symptoms. First, we defined adults as food insecure if they reported the following statements were either often or sometimes true.^1^ We worried our food would run out before we got money to buy more ^2^; and the food that we bought just didn't last and we didn't have money to get more. In addition, respondents were categorized as food insecure if they received or applied for income assistance from a food pantry or the Supplemental Nutrition Assistance Program (SNAP) in the past 7 days.

We defined financial hardship using the following question: “Suppose you have an unexpected expense that costs $400. Based on your current financial situation, how would you pay for this expense?” Respondents who chose the following options were categorized as experiencing financial hardship: borrow from a friend or family member; use a payday loan, deposit advance or overdraft; sell something; or I would not be able to pay for it right now. All answer options are summarized in Table 2.

Next, to evaluate mental health symptoms, participants were asked to report symptoms of anxiety, depression, loneliness, and hopelessness in the 7 days before survey administration. Participants were able to choose from the following list of options for each mental health symptom: not at all or <1 day, 1–2 days, 3–4 days, and 5–7 days. For multivariable models, we created binary variables for each mental health symptom and categorized those who responded as not at all or <1 day as zero, and recombined the other categories.

### Covariates

The following covariates were included in the multivariable analyses: age (18–29, 30–44, 25–59, 60+), gender (male/female), marital status (married/living with a partner, widowed/divorced/separated, never married), education categories (no high school diploma, HS graduate or equivalent, some college, baccalaureate degree or above), employment status (employed/unemployed), household income (<$50,000, $50,000–<$100,000, ≥$100,000), population density (rural, suburban, urban), census region (Northeast, Midwest, South, West), and insurance type (purchased plan/employer-sponsored/TRICARE/Medicaid/Medicare/Dually eligible/Veteran's Affairs benefits/uninsured).

### Data analysis

Descriptive statistics were summarized, by AA ethnic categories, as well as the total population and all AAs homogeneously presented. We include these comparisons to underscore the disparities that may exist within disaggregated AA ethnic groups. We used chi-square or exact tests to compare the prevalence of financial hardship and FI among AA ethnicities. To identify demographic groups that may be more likely to report FI, we estimated determinants of FI among AAs, including financial hardship and AA ethnic groups. We computed prevalence ratios with Poisson regression using robust estimation of standard errors.^15–17^ Potential variables for inclusion in the model were assessed using available sociodemographic variables and bivariate Poisson regression analysis.

Due to the exploratory nature of this analysis using a predictive framework, a *p*-value of <0.10 based on unadjusted regression results was used as a criterion to include the variable in the multivariable Poisson regression model. For multivariable Poisson regression models, adjusted prevalence ratios (aPR), and 95% confidence intervals (CIs) for each independent variable were calculated. We also used multivariable Poisson regression to estimate associations of any mental health symptom (≥1 day per week) with FI after adjustment for age group, sex, and income. Based on the exploratory nature of this analysis, we did not include an adjustment for multiple comparisons.^18,19^ All statistical analyses were conducted using Stata IC 15 (StataCorp LLC, College Station, TX). Sampling weights were applied to provide results that were nationally representative of the U.S. adult population.

## Results

In this sample of 10,760 adults (*weighted n*=418,209,893), 5.1% (*weighted n*=21,143,079; *unweighted n*=312) self-identified as an Asian ethnic group. AA adults were more likely to be male (66.4%) and these proportions differed by AA ethnic group (Table 1). By age group, 68.3% of AA adults were 18–44 years old. The greater proportion of younger adults among AAs was driven predominantly by South Asians (85.8%) and Filipino/Vietnamese (73.5%) adults who were more likely to be ≤44 years of age compared with their Chinese (48.6%) and Japanese/Korean (43.1%) peers. For educational attainment, 66.4% of all AAs reported at least a bachelor's degree; across the AA ethnic group, 66–75.4% adults reported at least a college degree. By marital status, Japanese/Korean American (81.9%) adults were more likely to report being married/cohabitating compared with South Asian (56.6%), Chinese (58.4%), and Filipino/Vietnamese (59.6%) American adults.

**Table 1.** Sociodemographic Characteristics Among the Study Population Overall by Asian American Ethnic Group, COVID-19 Household Impact Survey Respondents, April–June 2020

	Total		Asian		Chinese		South Asian		Filipino/Vietnamese		Japanese/Korean		p^*^
Unweighted *n*	10,760		312		84		77		57		43		
Weighted *n*	418,209,893		21,143,079		6,484,767		5,439,996		3,529,420		2,108,210		
	**%**	**95% CI**	**%**	**95% CI**	**%**	**95% CI**	**%**	**95% CI**	**%**	**95% CI**	**%**	**95% CI**	
Sex (male)	48.3	47.0–49.6	66.4	59.1–72.9	75.3	62.7–84.7	70.8	55.1–82.7	62.4	46.0–76.3	51.3	34.9–67.5	<0.001
Age groups													<0.001
18–44	46.0	19.3–21.8	68.3	61.2–74.6	48.6	35.7–61.6	85.8	73.4–93.0	73.5	58.3–84.6	43.1	27.5–60.1	
45+	54.0	23.2–25.4	31.7	25.4–38.8	51.4	38.4–64.3	14.2	7.0–26.6	26.5	15.4–41.7	56.9	39.9–72.5	
Education													<0.001
No HS diploma	9.8	8.8–10.8	2.5	1.1–5.5	0.0		0.0		9.6	3.6–23.3	0.0		
HS/GED	28.2	27.0–29.6	18.7	12.3–27.4	11.5	4.4–26.9	24.0	12.1–42.0	10.9	4.0–26.9	0.0		
Some college	27.7	26.7–28.7	12.5	8.8–17.3	13.1	7.0–23.3	5.3	1.6–16.5	13.4	6.0–27.1	28.0	15.5–45.2	
BA or above	34.3	33.1–35.5	66.4	58.4–73.5	75.4	61.5–85.4	70.7	53.5–83.4	66.0	49.8–79.3	72.0	54.8–84.5	
Marital status													<0.001
Married/cohabituating	57.2	55.9–58.5	56.6	49.0–63.9	58.4	45.0–70.6	56.6	41.6–70.4	59.6	42.8–74.5	81.9	68.0–90.6	
Widowed/divorced/separated	18.5	17.5–19.4	5.2	3.1–8.6	2.0	0.6–6.2	3.8	1.0–13.4	0.0		6.3	1.6–21.0	
Never married	24.4	23.2–25.6	38.2	31.0–46.0	39.7	27.6–53.2	39.5	26.0–54.9	40.4	25.5–57.2	11.8	5.8–22.8	
Population density													<0.001
Rural	9.1	8.4–9.8	0.6	0.1–2.4	0.0		0.0		0.0		6.0	1.5–21.1	
Suburban	18.8	17.8–19.7	3.7	1.6–8.4	6.7	1.7–22.7	1.0	0.3–3.3	2.9	0.4–17.9	0.2	0.0–1.6	
Urban	72.2	71.0–73.3	95.7	91.1–97.9	93.3	77.3–98.3	99.0	96.7–99.7	97.1	82.1–99.6	93.7	79.0–98.4	
Census region													<0.001
Northeast	17.4	16.4–18.5	18.4	13.5–24.7	21.9	12.6–35.3	32.1	20.3–46.7	0.0		21.7	10.6–39.4	
Midwest	20.7	19.8–21.7	8.2	5.1–13.0	11.2	4.5–25.2	5.7	2.7–11.5	12.6	5.0–28.2	3.7	1.5–9.0	
South	38.0	36.7–39.3	29.3	22.9–36.8	21.2	12.4–33.9	47.0	32.8–61.8	17.0	9.2–29.4	6.2	2.1–17.0	
West	23.8	22.8–24.9	44.0	36.8–51.4	45.7	33.1–58.9	15.2	8.7–25.3	70.3	55.0–82.2	68.4	51.5–81.5	
Employed (yes)	49.7	48.4–51.1	63.9	56.5–70.7	47.6	34.9–60.5	78.7	63.4–88.8	57.8	42.1–72.0	60.0	43.1–74.9	0.001
Annual household income													<0.001
<$50,000	45.8	44.5–47.1	25.2	19.0–32.7	27.2	17.1–40.5	6.3	2.0–17.9	36.0	21.5–53.6	33.8	19.9–51.4	
$50,000–<$100,000	32.1	30.9–33.3	36.0	29.4–43.2	26.9	16.2–41.4	30.0	19.0–44.0	45.3	30.3–61.2	39.8	25.2–56.4	
$100,000 and above	22.1	21.1–23.2	38.8	31.9–46.1	45.8	33.2–59.0	63.7	49.4–76.0	18.7	9.7–33.0	26.4	13.8–44.6	
Insurance type													
Self-purchased	17.4	16.4–18.5	22.8	16.9–29.9	35.8	23.8–49.9	10.9	5.0–22.0	29.2	15.6–48.0	13.5	5.5–29.4	0.005
Through employer	51.7	50.3–53.0	75.9	69.6–81.3	69.9	56.8–80.5	82.8	68.6–91.4	60.0	44.2–74.0	78.3	61.4–89.1	<0.001
Medicaid	23.5	22.4–24.7	12.9	9.0–18.2	14.1	7.2–25.9	10.5	4.2–24.1	21.7	11.5–37.2	16.9	8.0–32.2	<0.001
Medicare	25.3	24.2–26.4	6.7	4.2–10.4	4.4	1.8–10.6	6.7	2.4–17.1	9.7	4.4–19.9	7.3	2.9–17.4	<0.001
Uninsured	8.8	8.1–9.6	2.2	1.1–4.3	3.8	1.3–10.1	0.0		2.0	0.5–7.8	0.0		<0.001
Any mental health conditions in the past 7 days													
Felt anxiety	38.2	36.9–39.5	29.3	23.7–37.1	27.4	17.1–41.0	38.7	25.6–53.7	25.3	14.6–40.2	28.9	16.1–46.3	0.17
Felt depressed	34.4	32.9–35.9	35.3	28.5–42.8	29.2	18.6–42.6	43.9	29.8–42.6	28.7	17.3–43.6	27.3	15.1–44.3	0.42
Felt lonely	39.1	37.8–40.–4	37.7	30.9–45.0	41.8	29.6–55.1	35.5	22.6–50.8	22.9	13.0–37.4	27.9	14.7–46.4	0.19
Felt hopeless	38.9	37.7–40.3	36.5	29.8–43.7	35.3	24.3–48.1	40.7	26.9–56.1	37.4	23.8–53.5	24.5	12.4–42.6	0.82

^*^ *p*-value is based on a chi-square test comparing each Asian racial subcategory.

CI, confidence interval.

AA adults predominantly reported residing in urban settings (93.3–99%). In terms of annual household income, 25.2% of AA adults reported incomes of <$50,000, but within the AA groups, 36% of Filipino/Vietnamese and 33.8% of Japanese/Korean Americans reported incomes of <$50,000 compared with 27.2% of Chinese and 6.3% of South AAs. Overall, South Asian adults were most likely to report feeling anxious (38.7%), depressed (43.9%), and hopeless (40.7%) in the past 7 days compared with other AA ethnic groups. Chinese Americans were most likely to report feeling lonely (41.8%).

Overall, within the aggregated Asian ethnic group, 33.7% were categorized as food insecure (Table 2). However, when disaggregated, there were statistically significant differences in FI across Asian ethnic groups (*p*-value=0.011). Japanese/Korean adults reported the lowest prevalence of recent FI (13.9%, 95% CI 6.2–28.4). Among Filipino/Vietnamese adults, recent FI was highest at 52.9% (95% CI: 36.9–68.3). When disaggregated by Asian ethnic group, South Asian (6.8% and 22.5%, respectively) and Filipino/Vietnamese adults (11.3% and 32.3%, respectively) were more likely to report “often” and “sometimes” worrying about food running out compared with their Chinese and Japanese/Korean adults (*p*=0.01).

**Table 2.** Disparities in Financial Hardship and Food Insecurity Measurement Items Among Asian American Respondents of the COVID-19 Household Impact Survey

	Total		NH Asian		Chinese		Asian Indian		Filipino/Vietnamese		Japanese/Korean		
Unweighted *n*	10,760		312		84		77		57		43		
Weighted *n*	418,209,893		21,143,079		6,484,767		5,439,996		3,529,420		2,108,210		
	**Col %**	**95% CI**	**Col %**	**95% CI**	**Col %**	**95% CI**	**Col %**	**95% CI**	**Col %**	**95% CI**	**Col %**	**95% CI**	***p*** ^a^
FI													
Overall prevalence	33.8	32.6–35.1	33.7	26.7–41.5	25.6	15.5–39.8	29.7	17.6–45.5	52.9	36.9–68.3	13.9	6.2–28.4	0.01
Food insecurity measures													
We worried our food would run out before we got money to buy more.													0.011
Often true^b^	6.2	5.5–6.9	5.8	3.2–10.2	5.1	1.4–16.4	6.8	2.2–19.2	11.3	4.3–26.7	0.0		
Sometimes true	20.3	19.1–21.4	21.0	15.0–28.7	7.4	3.1–16.6	22.5	11.8–38.6	32.3	18.4–50.1	6.4	2.0–18.4	
Never true	73.6	72.3–74.8	73.2	65.4–79.7	87.5	76.2–93.9	70.7	54.8–82.8	56.4	39.7–71.7	93.6	81.6–98.0	
The food that we bought just didn't last, and we didn't have money to get more.													0.037
Often true	4.5	4.0–5.1	4.8	2.6–8.6	2.6	0.8–8.1	5.8	1.6–19.2	11.3	4.3–26.7	0.0		
Sometimes true	16.9	15.9–18.0	20.2	14.2–28.0	9.9	4.4–21.0	19.6	9.7–35.8	28.7	15.4–47.1	7.9	3.0–19.3	
Never true	78.6	77.4–79.7	75.0	67.2–81.4	87.5	76.6–93.7	74.5	58.4–85.9	60.0	42.8–75.1	92.1	80.7–97.0	
In the past 7 days, have you either received, applied for any of the following forms of income assistance or not?													
SNAP													0.001
Received	10.7	10.0–11.5	2.3	1.2–4.3	0.5	0.1–3.6	1.0	0.3–3.3	5.8	1.9–16.3	0.0		
Applied for	2.2	1.8–2.8	2.5	1.0–6.2	2.1	0.5–8.0	5.2	1.2–19.5	3.1	0.5–17.2	0.0		
Tried to apply for	2.0	1.6–2.4	2.3	1.1–4.8	0.0		1.0	0.2–4.0	12.0	5.2–25.1	0.0		
Did not receive nor apply for any	85.1	84.1–86.0	92.9	89.0–95.5	97.4	91.9–99.2	92.8	80.7–97.5	79.1	64.5–88.7	100.0		
A food pantry													0.109
Received	6.8	6.1–7.4	5.3	2.6–10.5	9.6	3.4–24.1	3.9	0.6–21.4	3.5	0.9–13.3	6.0	1.5–21.3	
Applied for	0.9	0.7–1.2	0.8	0.3–1.7	0.0		2.5	0.9–6.4	0.0		0.0		
Tried to apply for	1.0	0.8–1.4	2.3	1.0–5.2	2.1	0.5–8.0	0.0		9.7	3.4–24.6	0.0		
Did not receive nor apply for any	91.3	90.5–92.0	91.7	86.5–95.0	88.4	74.6–95.2	93.6	80.8–98.1	86.7	72.1–94.3	94.0	78.7–98.5	
Financial hardship													
Overall prevalence	28.8	27.5–30.0	20.5	14.9–27.4	18.4	9.7–32.2	14.1	5.7–30.6	24.5	13.6–40.1	8.6	2.8–23.8	0.019
Financial hardship measure													
Suppose that you have an unexpected expense that costs $400. Based on your current financial situation, how would you pay for this expense? If you would use more than one method to cover this expense, please select all that apply													
Put it on my credit card and pay it off in full at the next statement	34.0	32.7–35.2	69.3	61.7–75.9	83.5	72.7–90.5	72.5	55.3–84.9	66.5	50.6–79.3	65.5	47.7–79.8	0.176
Put it on my credit card and pay it off over time	18.5	17.6–19.6	17.4	12.5–23.7	19.4	10.3–33.3	19.5	9.4–36.2	15.5	8.0–28.0	14.8	6.3–30.8	0.91
Use money currently in my checking or savings account or with cash	50.7	49.4–52.0	42.6	35.4–50.1	40.8	28.5–54.4	36.4	23.7–51.4	30.4	18.7–45.5	51.3	34.8–67.6	0.411
Use money from a bank loan or line of credit	3.0	2.6–3.4	2.6	1.4–4.9	4.3	1.4–12.0	1.0	0.2–4.0	0.0	0	1.9	0.7–4.7	0.169
Borrow from a friend or family member	9.3	8.5–10.2	11.8	7.6–17.8	13.3	6.0–26.9	0.4	0.1–2.7	18.2	8.8–33.8	8.6	2.7–23.9	0.009
Use a payday loan, deposit advance or overdraft	1.9	1.5–2.2	0.7	0.2–2.0	0.5	0.1–3.6	0.0		0.0		0.2	0.0–1.3	0.82
Sell something	7.0	6.3–7.7	7.3	4.0–12.9	5.1	1.4–16.4	4.8	1.3–16.8	6.3	2.2–17.0	6.9	1.7–23.8	0.973
I would not be able to pay for it right now	15.5	14.5–16.5	4.3	1.9–9.3	0.0		8.8	2.5–27.2	0.0	0	2.7	0.7–9.6	0.04

^a^ *p*-value is based on chi-square or exact test comparing across Asian American ethnic groups.

^b^ Italicized answer options/statements were included in food insecurity and financial hardship definitions.

FI, food insecurity; NH, non-hispanic; SNAP, Supplemental Nutrition Assistance Program.

Filipino/Vietnamese adults were also more likely (11.3% and 28.7%, respectively) to report “The food we bought just didn't last and we didn't have money to get more” being “often true” and “sometimes true” compared with all other Asian ethnic groups (*p*=0.037). Filipino/Vietnamese adults were more likely to report receiving (5.8%), applying for (3.1%), and trying to apply (12.0%) for SNAP compared with all other Asian ethnic groups (*p*<0.001). The prevalence of financial hardship among AA groups was 20.5%, but was disparate across AA ethnic groups. Filipino and Vietnamese adults had the highest prevalence of financial hardship (24.5%), whereas Japanese and Korean adults had the lowest (8.6%) (*p*=0.019).

Similarly, when asked how they would handle an unexpected expense that costs $400, Filipino and Vietnamese adults were most likely to report they would borrow from a friend or family member (18.2%) compared with other AA ethnic groups (*p*=0.009). South Asians were most likely to report they would not be able to pay for it right now (8.8%) compared with other ethnic groups (*p*=0.04) (Table 2).

Table 3 summarizes determinants of FI among AA adults. We observed that compared with Chinese adults, Filipino and Vietnamese adults (aPR: 2.81, 95% CI: 1.49–5.29) were more likely to experience FI. In unadjusted analyses, financial hardship led to a 124% higher prevalence of FI among AAs. AA adults who were widowed/divorced/married were more likely to experience FI compared with those who were married or living with a partner (aPR: 3.14, 95% CI: 1.37–7.23). AA adults with only a high school degree were more likely to experience FI (aPR: 3.46, 95% CI: 1.96–6.11) compared with their counterparts with a baccalaureate degree. AAs residing in rural areas were more likely to experience FI (aPR: 7.65, 95% CI: 1.17–50.14).

**Table 3.** Determinants of Food Insecurity Among Asian American Respondents of the COVID-19 Household Impact Survey (Unweighted ***n*=312**)

	Unadjusted PR	95% CI	Adjusted PR	95% CI
AA ethnic group				
Chinese	Ref.		Ref.	
South Asian	1.15	0.59–2.25	1.34	0.74–2.45
Filipino+Vietnamese	2.05	1.16–3.59	2.81	1.49–5.29
Japanese+Korean	0.54	0.22–1.33	0.58	0.17–2.02
Other Asian	1.81	0.93–3.53	0.97	0.54–1.74
Financial hardship	2.46	1.63–3.72	1.24	0.60–2.55
Age				
18–29	1.37	0.74–2.54	1.99	0.23–6.31
30–44	0.95	0.53–1.72	2.78	0.53–14.71
45–49	0.27	0.09–0.75	0.97	0.15–6.34
60+	Ref.		Ref.	
Sex				
Male	Ref.		Ref.	
Female	0.48	0.29–0.78	0.58	0.33–1.00
Marital status				
Married/living with partner	Ref.		Ref.	
Widowed/divorced/separated	2.60	1.54–4.40	3.14	1.37–7.23
Never married	1.99	1.28–3.07	1.44	0.82–2.54
Insurance type				
Purchased plan	0.91	0.52–1.59	—	
Employer-sponsored	0.74	0.48–1.13	—	
TRICARE	1.85	1.05–3.26	0.94	0.37–2.40
Medicaid	2.19	1.47–3.23	1.39	0.57–3.35
Medicare	1.75	1.09–2.82	1.85	0.41–8.30
Dually eligible (Medicare and Medicaid)†	2.19	1.32–3.66	0.78	0.10–6.44
VA	3.00	2.39–3.76	1.89	0.41–8.74
No insurance	1.10	0.46–2.63	—	
Employment status				
Employed/self-employed	0.91	0.58–1.42	—	
Education				
No HS diploma	1.24	0.36–4.29	0.24	0.04–1.31
HS graduate	2.76	1.77–4.30	3.46	1.96–6.11
Some college	1.81	1.09–3.01	1.05	0.55–1.99
Baccalaureate or above	Ref.		Ref.	
Household income				
<$30,000	3.78	2.08–6.86	2.54	1.27–5.06
$30,000–<$50,000	2.20	1.06–4.53	2.01	0.74–5.48
$50,000–<$75,000	1.84	0.91–3.74	1.11	0.50–2.44
$75,000–<$100,000	1.85	0.91–3.74	1.76	1.00–3.10
≥$100,000	Ref.		Ref.	
Region			**—**	
Northeast	Ref.			
Midwest	0.76	0.31–1.84		
South	0.71	0.36–1.41		
West	1.14	0.66–1.98		
Population density				
Rural	2.98	2.37–3.75	7.65	1.17–50.14
Suburban	0.83	0.26–2.64	0.8	0.33–1.96
Urban	Ref			

AA, Asian America; PR, prevalence ratio; VA, Veteran's Affairs benefits.

**FIG. 1.**
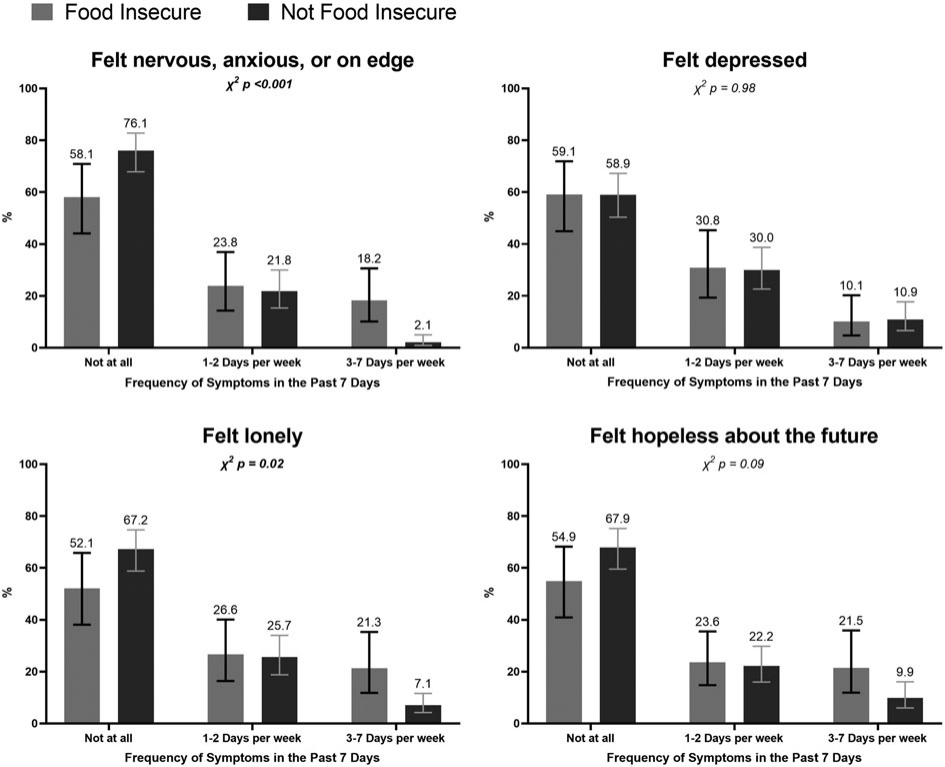
Prevalence of mental health symptoms among food-insecure AAs (April–June 2020) (unweighted *n*=312). AAs, Asian Americans.

**FIG. 2.**
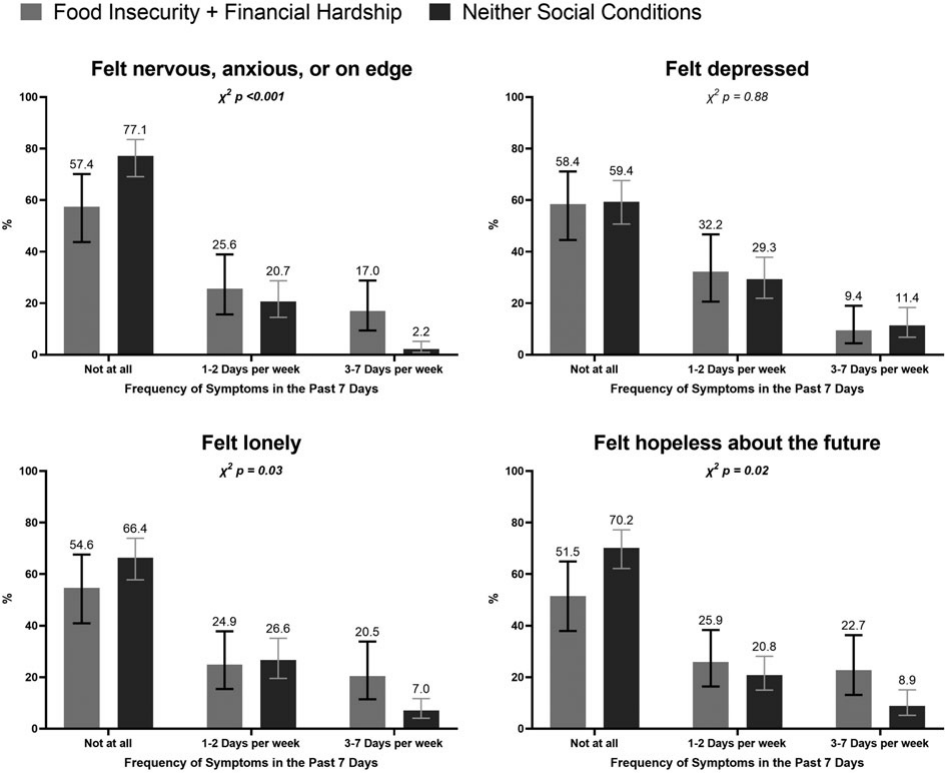
Prevalence of mental health symptoms among AA adults experiencing FI and financial hardship (April–June 2020) (unweighted *n*=312). FI, food insecurity.

AA adults experiencing FI were more likely to report feeling nervous, anxious, or on edge 3–7 days per week (18%) compared with those without FI (2%) (*p*<0.001). Similarly, food-insecure AA adults were more likely to report feeling lonely (21% vs. 7%, *p*=0.02) at least 3–7 days per week (Fig. 1). AA adults who experienced both FI and financial hardship were more likely to report feeling nervous, anxious, or on edge (17% vs. 2%, *p*<0.001), feeling lonely (21% vs. 7%, *p*=0.03), and feeling hopeless about the future (23% vs. 9%, *p*=0.02) 307 days per week (Fig. 2).

On multivariable analyses, after adjustment for age group, sex, and income, we found that those who were food insecure experienced 38% higher prevalence of feelings of anxiety (aPR: 1.38, 95% CI: 1.29–1.50) and depression (aPR: 1.38, 95% CI: 1.28–1.49). In addition, AA adults who experienced FI were more likely to experience feelings of loneliness (aPR: 1.43, 95% CI: 1.33–1.54) and hopelessness (aPR: 1.41, 95% CI: 1.32–1.52) compared with those who are not food insecure.

## Discussion

Overall, our results suggest that about one in three of AA adults reported experiencing FI and one in five experienced financial hardship during the COVID-19 pandemic. We observed important disparities among AA adults: although one-third of AAs reported experiencing FI overall, about half of Filipino and Vietnamese adults experienced FI, which is comparable with the prevalence of FI reported among non-hispanic (NH)-Black adults based on national data collected early in the pandemic.^4^ These disparities would be missed by grouping all AA adults into one homogenous group. We also demonstrate that among AAs reporting FI and financial hardship, feelings of anxiety and hopelessness were prevalent.

Household FI is a leading nutritional issue in the United States characterized by disparities based on race/ethnicity and social determinants of health. In 2018, a nationally representative survey from the USDA reported that about 1 in 10 of U.S. households—14.3 million households were food insecure.^20^ Within the United States, there is significant heterogeneity in the burden of FI. For example, in 2018, 21.2% of Black and 16.2% of Hispanic or Latinx households experienced FI compared with the national average of 11.1%.^1^ The disproportionate burden of FI among minoritized communities in the United States is directly related to poverty, financial hardship, low/unemployment, and disability.^21^ During the COVID-19 pandemic, FI has been exacerbated by economic disruptions, loss of employment, and financial hardships.

In fact, early data from the 2020 U.S. Census Household Pulse Survey show that the prevalence of FI before March 2020 was 30% but increased to 43% by late April 2020 among responding households.^12^ The salience of FI has been underscored due to its documented associations with negative health outcomes, including poor cognitive development,^22^ poor dietary choices,^23^ and mental illness.^24^ For example, experiences of hunger due to FI were highly associated with serious psychological distress among Black adults.^25^ Our study demonstrated not only a high prevalence of FI among specific AA ethnic groups, but also showed that respondents who experience FI were more likely to report frequent anxiety and hopelessness symptoms. We were unable to conduct disaggregated multivariable analyses to investigate the role of FI and financial hardship within specific AA ethnic groups.

Nonetheless, given that Filipino and Vietnamese adults experience both the highest prevalence of FI and financial hardship, it is important to ensure that AA ethnic groups are addressing their mental health symptoms potentially stemming from their disproportionate financial constraints.

Prior research conducted on FI among AAs has found that Vietnamese and Filipino adults experience high levels of FI due to financial hardship, similar to our findings. A report on diet changes and food shopping behaviors among AAs during the COVID-19 pandemic established that a higher percentage of Filipino and Vietnamese adults suffered from economic hardships that negatively impacted their food security.^26^ In addition, a California-specific survey conducted before the pandemic found the highest prevalence of FI to be among Vietnamese adults and the lowest among Japanese.^27^

This study found that FI among AA subgroups was associated with lower acculturation, as measured by the prevalence of speaking only a foreign language at home and country of birth. In this survey of Californian AAs, the Vietnamese American population had the highest prevalence of speaking only a foreign language at home (52%) and the highest number of foreign-born individuals (88%) compared with the Japanese American population, with 5% and 27% speaking a foreign language at home and number of foreign-born individuals, respectively. In addition, although SNAP participation overall among AAs is low, the highest rate of SNAP participation was among Vietnamese Americans who spoke a non-English language only (15%) and were foreign born (14%, 27). A major limitation of this analysis is the unavailability of English language preferences and nativity. Future work to evaluate the socioeconomic impacts of the pandemic on AAs should include these measurements.

FI, and associated financial hardship, is an important public health issue due to the potential downstream adverse effects of poor nutrition on chronic disease prevention. In terms of food consumption and nutritional intake, FI is strongly associated with a low Healthy Eating Index score among all races/ethnicities.^28^ Specifically, FI is associated with lower scores on protein intake, but higher scores on food items with added sugars among whites. Among AAs, FI is associated with a lower score on fresh fruit consumption.^28^ Poor eating habits among AAs during the pandemic have been exacerbated during the pandemic due to the rise of xenophobia and structural racism.^29^ For example, a study of food shopping behaviors and dietary changes during the pandemic revealed that South Asian and southeast Asian (Filipino/Vietnamese) Americans reported more concerns with receiving adequate food resources in comparison with East Asian (Japanese/Koreans) Americans.^26^

Southeast Asians also reported more economic challenges, whereas South Asians more frequently reported obstacles with access to food and getting to the food store. This study demonstrated a potential driver of the shift in food shopping behaviors may be heightened fear associated with going out to buy food during the COVID-19 pandemic among AA households compared wiht white households. In addition, barriers such as lack of transportation, immobility, or health issues may be further related to the increased likelihood of FI in Vietnamese and Filipino households.^26^

Our study adds to the established literature demonstrating the role of FI and financial hardship within the context of mental health. Prior research has demonstrated that experiencing FI over a period of 12 months increases the likelihood of having clinical depression among AAs, but the magnitude of the association between FI and depression can vary across AA subgroups.^30^ For example, one prior national study using data from the National Latino and AAs Study National Latino and AAs Study demonstrated that Filipino Americans experienced the highest rates of FI (41%) compared with all other Asians (38%), Chinese Americans (26%), and Vietnamese Americans (26%, 30). However, the association between FI and depression was highest among the Chinese Americans and all other Asian subgroups, and lowest among the Vietnamese and Filipino adults.^30^ Again, failure to disaggregate data by AA ethnic group would mask the differences in mental health burdens associated with FI across these AA groups.

These findings, for each of the distinct AA ethnic groups noted above, are consistent with prior literature showing that FI is associated with a higher prevalence of mental health burdens. In addition, these findings underscore the need for creating and connecting culturally appropriate and relevant mental health services for AAs. Moreover, given the disparities in economic hardship across these groups, it is important to ensure that there is access to affordable mental health care and treatment services. The short- and long-term ramifications of the COVID-19 pandemic on FI, mental health, and well-being among AA ethnic groups require careful and concerted prevention and intervention efforts. Future research should focus on evaluating reasons for the association observed between FI and poor mental health, particularly in the context of AA social experiences.

The findings of this study should be interpreted within the context of several limitations. First, although the data are collected over three time points, they are cross-sectional in nature. Therefore, we are unable to ascertain the temporality of each socioeconomic hardship, including FI and financial hardship, and mental health symptoms. In addition, data regarding each outcome before the pandemic are unavailable, and we are unable to demonstrate the role of the pandemic on FI and financial hardship among AAs. Although the survey sample was weighted to reflect nationally representative estimates, our AA sample size was small and was unable to demonstrate associations of mental health symptoms with FI and financial hardship disaggregated by the AA group.

Similar to prior national surveys and health research,^31–33^ the COVID-19 Household Survey respondent population only included 3% AA adults, which is an underrepresentation of those living in the United States; AAs account for 5.7% of the nation's population according to estimates from the 2019 Census Bureau. It is important to note that the large majority of AA respondents were between the ages of 18–44 years, suggesting that barriers existed to recruiting older AA participants. Prior studies have documented the barriers to recruiting AAs to research studies in the United States, such as limitations in accessibility to internet-based surveys, limited data collection in Asian languages, and uneven distribution of geographic representation of participants.^33^ One significant strategy to improve recruitment of elderly AA adults includes endorsement of research from a trusted and known individual, such as a family member.^34–36^ Future national surveys should use recruitment methods involving family members and social networks.

In conclusion, our study suggests that AAs who experience FI may be experiencing higher mental health symptoms during the COVID-19 pandemic. Given the economic impact of the pandemic on employment and financial stability among AAs,^37^ future interventions to improve mental health outcomes among this vulnerable population should be prioritized. Our research underscores the significance of developing culturally and linguistically appropriate resources to curtail the financial impacts of the pandemic on racial/ethnic minorities in the United States. By providing these AA ethnic group insights, community-based organizations working in diverse AA communities may optimize their efforts to address both the socioeconomic and mental health needs of this diverse community.

## Abbreviations Used

AA: Asian American
aPR: adjusted prevalence ratio
CI: confidence interval
FI: food insecurity
NH: non-hispanic
NORC: National Opinion Research Center
SNAP: Supplemental Nutrition Assistance Program
USDA: United States Department of Agriculture
VA: Veteran's Affairs
